# Recent Advances in Videolaryngoscopy for One-Lung Ventilation in Thoracic Anesthesia: A Narrative Review

**DOI:** 10.3389/fmed.2022.822646

**Published:** 2022-06-13

**Authors:** Wenlong Yao, Meihong Li, Chuanhan Zhang, Ailin Luo

**Affiliations:** Department of Anesthesiology, Tongji Hospital, Tongji Medical College, Huazhong University of Science and Technology, Wuhan, China

**Keywords:** videolaryngoscope, one-lung ventilation, double-lumen tube, bronchial blocker, difficult airways

## Abstract

Since their advent, videolaryngoscopes have played an important role in various types of airway management. Lung isolation techniques are often required for thoracic surgery to achieve one-lung ventilation with a double-lumen tube (DLT) or bronchial blocker (BB). In the case of difficult airways, one-lung ventilation is extremely challenging. The purpose of this review is to identify the roles of videolaryngoscopes in thoracic airway management, including normal and difficult airways. Extensive literature related to videolaryngoscopy and one-lung ventilation was analyzed. We summarized videolaryngoscope-guided DLT intubation techniques and discussed the roles of videolaryngoscopy in DLT intubation in normal airways by comparison with direct laryngoscopy. The different types of videolaryngoscopes for DLT intubation are also compared. In addition, we highlighted several strategies to achieve one-lung ventilation in difficult airways using videolaryngoscopes. A non-channeled or channeled videolaryngoscope is suitable for DLT intubation. It can improve glottis exposure and increase the success rate at the first attempt, but it has no advantage in saving intubation time and increases the incidence of DLT mispositioning. Thus, it is not considered as the first choice for patients with anticipated normal airways. Current evidence did not indicate the superiority of any videolaryngoscope to another for DLT intubation. The choice of videolaryngoscope is based on individual experience, preference, and availability. For patients with difficult airways, videolaryngoscope-guided DLT intubation is a primary and effective method. In case of failure, videolaryngoscope-guided single-lumen tube (SLT) intubation can often be achieved or combined with the aid of fibreoptic bronchoscopy. Placement of a DLT over an airway exchange catheter, inserting a BB *via* an SLT, or capnothorax can be selected for lung isolation.

## Introduction

In thoracic anesthesia, lung isolation techniques are often required to achieve one-lung ventilation. Double-lumen tubes (DLTs) are widely used in adult thoracic surgery because they provide reliable isolation and separate ventilation. However, owing to their large diameter, high rigidity, and complex configuration, it is not easy to intubate a DLT compared with a routine endotracheal tube (ETT); this difficulty is increased in the case of a difficult airway (1–3). In such cases, it is necessary to secure the airway and maintain oxygenation, and then consider the possibility of lung collapse (2, 3).

Since their advent, videolaryngoscopes have played an important role in airway management, including expected and unexpected difficult airways (4, 5). There are many reports on using videolaryngoscopes in DLT intubation, but their advantages in thoracic anesthesia are not as distinct as in other specialties (6–8). This review summarizes the techniques of videolaryngoscope-guided DLT intubation and discusses the roles of videolaryngoscopy in DLT intubation in normal airways compared with direct laryngoscopy. The different types of videolaryngoscopes for DLT intubation are also compared. In addition, we highlight several strategies to achieve one-lung ventilation in difficult airways using videolaryngoscopes.

## Overview of Videolaryngoscopy

Videolaryngoscopy is a new type of laryngoscopy that incorporates video systems using micro-camera technology and optical or fiber optical guided transmission (9). GlideScope, invented by John A. Pacey, was the first videolaryngoscope and was officially introduced into clinical practice in 2001 (10). It was first published for resolving difficult intubation in 2003 (11). Various videolaryngoscopes have been developed since 2006. Most videolaryngoscopes contain a light source and an image sensor close to the blade tip. Instead of line of sight with a direct laryngoscope, the videolaryngoscope does not require alignment of the oral, pharyngeal, and laryngeal axes and enables the operator to visualize the glottis on the video screen. Videolaryngoscopy has several advantages over direct laryngoscopy (12, 13), such as a better view of the larynx, increased success rate of intubation, short intubation time, and less force required for intubation (14, 15). The videolaryngoscopy technique is also easy to learn especially for novices (16, 17).

In the past 20 years, videolaryngoscopy has played important roles in airway management (18). It has not only managed expected difficult airways (13, 19), such as in the case of morbid obesity, limited mouth opening, cervical immobility, and oropharyngeal masses, but also unexpected difficult airways (20). This method has also been successfully applied in obstetric anesthesia (21), pediatric anesthesia (22), emergent intubation (23), nasotracheal intubation (24), and awake intubation (25). Therefore, guidelines for difficult airway management have indicated that videolaryngoscopy can be applied as an initial approach for difficult intubation (26) or a rescue technique for unanticipated difficult intubation (18), and all anesthetists should be proficient with the use of a videolaryngoscope (5).

**Figure 1:**
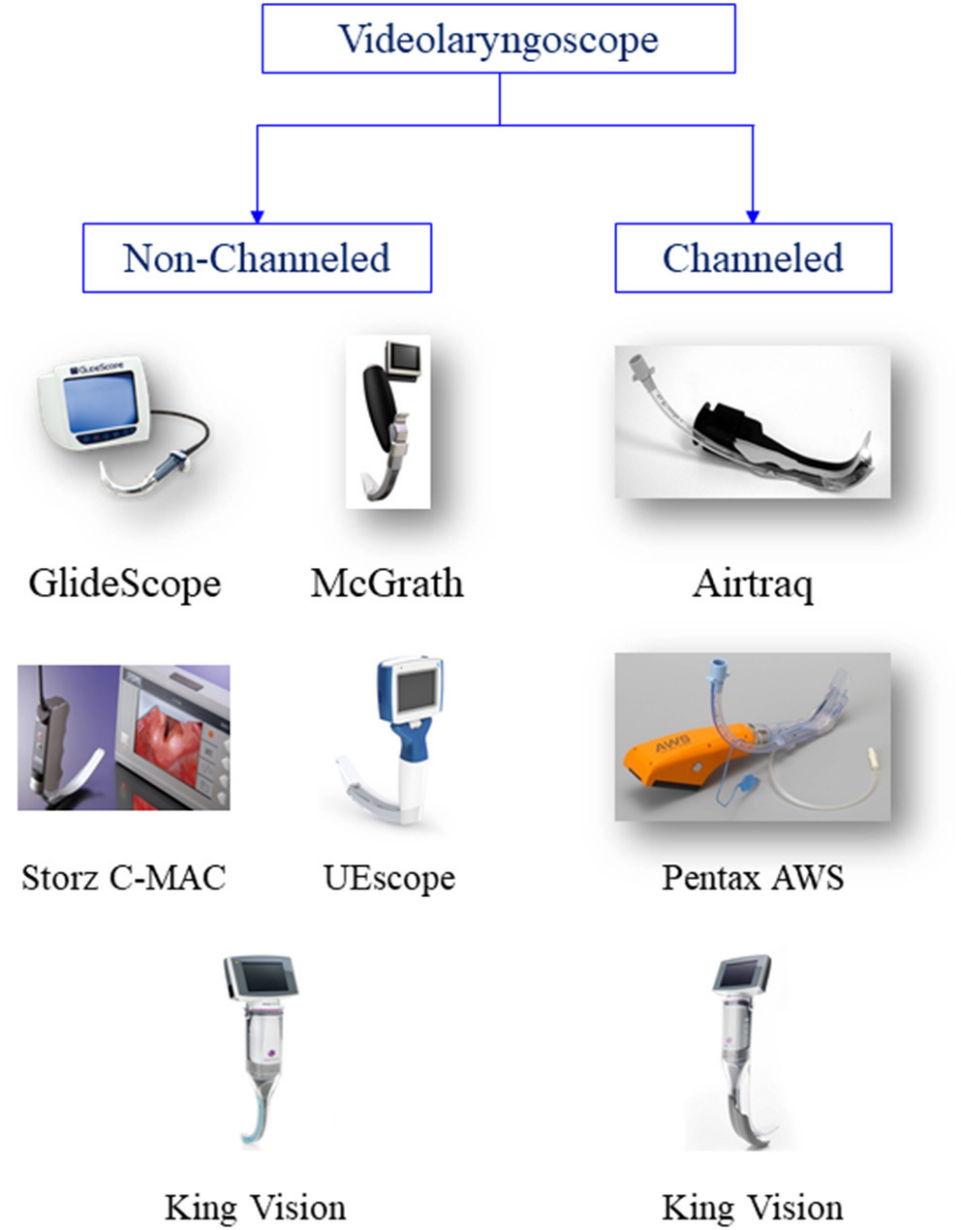
Classfication of videolaryngoscopes. GlideScope image courtesy of Verathon, USA. McGrath series 5 image courtesy of Aircraft Medical, UK. Airtraq image courtesy of Prodol Meditec, Spain. C-MAC image courtesy of KARL STORZ Endoscopy, Germany. UEscope image courtesy of UE Medical Corp, China. Pentax AWS, King Vision image courtesy of Ambu USA. Part of this figure is taken from Healy et al BMC Anesthesiol. 2012; 12: 32. ©2012 Healy et al.; licensee BioMed Central Ltd. Reproduced under the terms of its Creative Commons Attribution License (2.0).

Videolaryngoscopes can be classified as non-channeled or channeled. For example, GlideScope (Verathon Inc, Bothell, WA, USA) (27), McGrath (Aircraft Medical, Edinburgh, UK) (28), Storz (Karl Storz, Tuttlingen, Germany) (29), UEscope (UE Medical Corp, Zhejiang, China) (30), and non-channeled King Vision (Ambu A/S, Ballerup, Denmark) (31) are non-channeled videolaryngoscopes. They are similar to a standard laryngoscope (Figure 1). However, it may be difficult to place the tube into the glottis despite obtaining a good laryngeal view when using these devices (18). This difficulty can be resolved by a rigid stylet. A pre-shaped tracheal tube is recommended to facilitate intubation (10, 20). Channeled videolaryngoscopes, such as Airtraq (Prodol Meditec, Vizcaya, Spain) (32, 33), Pentax Airway Scope (Nihon Kohden, Tokyo, Japan) (34), and King Vision (Ambu A/S, Ballerup, Denmark) (35), have a guiding channel. The lubricated ETT is preloaded into the guiding channel. The videolaryngoscope is inserted to obtain a glottic view in the midline, and the tube is then placed into the trachea through the channel.

In addition, based on the blade angle, videolaryngoscopes can be classified as standard or hyperangulated (36). The Storz V-Mac (37), Storz C-Mac (38), and McGrath MAC devices were designed using a standard laryngoscope blade but with a camera incorporated into the distal tip. After insertion of the blade into the mouth, the glottis can be viewed alongside the blade following the traditional method or on the monitor. GlideScope, McGrath Series 5, Storz D-blade, and Airtraq have hyperangulated blades. They can increase the field and angle of view with less neck flexion and improve the glottic view compared with direct laryngoscopy (27, 39).

Videostylets, such as Optiscope, Trachway, Shikani optical stylet, and Light Wand, are other types of videoscope (36). They differ from traditional laryngoscopes in design and intubating techniques, and they are not considered the first rescue choice after failed intubation with a direct laryngoscope (18). Although they have extensive advantages in limited mouth opening and cervical fixation cases, they are outside the scope of this review.

## Key Points of Videolaryngoscope-Guided DLT Intubation

Many studies have reported the application of different types of videolaryngoscopes in DLT intubation, such as GlideScope (40, 41), McGrath (42), Airtraq (43, 44), CEL-100 (45), Pentax Airway Scope (46, 47), C-Mac D blade (48), King Vision (KVL) (49).

Generally, DLT intubation comprises four steps: glottis exposure using a videolaryngoscope, guiding the tip of the DLT into the glottis, advancing the tube through the glottis until it enters the appropriate main bronchus, and confirming the position of the DLT by fibreoptic bronchoscopy (FOB) (50). In the next section, we discuss GlideScope and Airtraq as typical examples of non-channeled and channeled videolaryngoscopes, respectively, to illustrate videolaryngoscope-guided DLT placement.

### GlideScope: A Non-channeled Videolaryngoscope

Since its introduction in 2001, GlideScope has been one of the most extensively studied videolaryngoscopes for DLT intubation. GlideScope provides superior glottis views to direct laryngoscopy; however, it can often be difficult to place the DLT into the glottis and advance the tube into the trachea when using GlideScope (40, 51) and other non-channeled videolaryngoscopes (7, 45). Several tips can be used to facilitate DLT intubation using GlideScope.

1. A pre-shaped DLT with a malleable stylet following the curve of the GlideScope should be used (40, 52). An appropriate stylet angle may lead to fewer failed intubation attempts and less airway trauma. Stylets with a 90° angle resulted in easier and faster intubation than those with a 60° angle using the GlideScope in single lumen tube (SLT) intubation (53, 54). Owing to the thick diameter of the DLT, manipulation inside the oropharyngeal space is restricted. Adjusting the DLT into the glottis was more difficult than the placement of the SLT. Hernandez and Wong (40) first described the successful use of GlideScope for DLT placement in a patient with a potentially difficult airway. They recommended bending the stylet of the DLT at 16–20 cm proximal to the tip to follow the curve of the GlideScope. Bustamante et al. (52) and Hsu et al. (41) recommended pre-curving the tube at the distal 10–12 cm of the DLT. The GlideRite DLT Stylet is a semi-rigid intubating stylet recommended by Bussieres et al. (55, 56). However, the optimal shape and angle of the stylet required for successful DLT intubation with GlideScope have not been determined in a randomized controlled study.
2. Sequentially rotate the tube to the desired depth when advancing the DLT. After the distal DLT passes through the vocal cord, difficulty can arise when attempting to advance it with GlideScope, because the distal concavity is directed anteriorly to the tracheal wall, and the axis of the bronchial lumen is nearly perpendicular to the axis of the trachea. Bustamante et al. (52) recommended that after the tip of the bronchial lumen engages in the glottis, the stylet should be removed, and the DLT should be rotated 180° counter-clockwise to facilitate passage of the bronchial cuff. An additional 90° clockwise rotation should then be performed to align the DLT with the left main bronchus. Hsu et al. (57) also reported a modified technique. After the DLT was inserted through the vocal cords, the DLT was advanced gently by rotating in a 90° clockwise direction until resistance was noted. The modified technique saves intubation time and reduces the severity of post-intubation complications, compared with the 180° clockwise rotation of the DLT during the placement of a DLT using GlideScope.
3. Sequential rotation could increase the risk of incorrect tube positioning during videolaryngoscope-guided DLT placement (58). For example, the tip of left-sided DLT could be migrated into the right bronchus. It is important to confirm the DLT position using FOB.
4. Take care to avoid the tracheal cuff scraping by the teeth during DLT insertion.

### Airtraq: A Channeled Videolaryngoscope

Hirabayashi and Seo (43) first reported the successful use of Airtraq for DLT intubation in 2007 and recommended Airtraq as an alternative approach for DLT placement.

The Airtraq videolaryngoscope with a guiding channel offers multiple options for visualizing the glottis, including a direct view, AWDR video system, A-360 Wi-Fi camera, universal adapter for smartphones, or Endo cam connection (59–61). The side channel can accommodate a tube with an external diameter of ≤ 19 mm, which allows for the placement of DLTs of 28–41 Fr. The inner surface of the side channel is treated with a concavo-convex pattern to reduce tube friction.

For Airtraq-guided DLT intubation, remove the original stylet, lubricate the DLT and channel, then preload the tube into the channel before intubation. Airtraq is inserted into the midline of the patient's mouth to slide it over the center of the tongue until the glottis structures are identified (16). Once the bronchial cuff passes through the glottis, the DLT is rotated 90° counter-clockwise and further advanced until resistance is felt. The tube is separated from the channel, and the laryngoscope is removed from the mouth (62, 63). Finally, the position of the DLT is checked using FOB. As the DLT was loaded in the channel during Airtraq-guided DLT intubation, one distinct advantage is that Airtraq has zero tracheal cuff rupture, compared with the Macintosh, GlideScope and non-channel KVL (64).

## Comparison of Videolaryngoscopy and Direct Laryngoscopy for DLT Intubation in Normal Airways

To date, 19 prospective randomized controlled studies have reported the efficacy of six videolaryngoscopes for DLT intubation compared with the Macintosh laryngoscope (Table 1) in patients with predicted normal airways. The main outcomes included glottic view, intubation time, success rate, intubation difficulty score, incidence of malposition, postoperative sore throat, hoarseness, related complications, and intubation-related stress response. Except that videolaryngoscopy can provide a better view of the glottis than the Macintosh, other findings were inconsistent between different studies. This heterogeneity may be attributed to the performer's experience, type of videolaryngoscope used, and primary outcome definition.

**Table 1:** Randomized controlled trials on videolaryngoscope vs. the Macintosh laryngoscope for double lumen tube intubation.

**Classification**	**Videolaryngoscope**	**Number of studies**	**References**
Non-Channeled	GlideScope	9	Bensghir et al. (65) Hsu et al. (41) Russell et al. (51) Yi et al. (66) El-Tahan et al. (67) Wei and Tian (68) El-Tahan et al. (64) Huang et al. (6) Risse et al. (69)
	McGrath	4	Kido et al. (70) Yao et al. (7) Yoo et al. (71) Bakshi et al. (72)
	CEL-100	1	Lin et al. (45)
	C-MAC D-blade	2	Shah et al. (48) Huang et al. (6)
	King Vision	2	El-Tahan et al. (67) El-Tahan et al. (64)
Channeled	Airtraq	6	Jiang et al. (73) Wasem et al. (74) Hamp et al. (75) El-Tahan et al. (67) El-Tahan et al. (64) Feng et al. (76)

Two studies demonstrated that GlideScope provided a shorter intubation time and a lower incidence of sore throat and hoarseness than the Macintosh laryngoscope (41, 65). In contrast, Russell et al. (51) reported that GlideScope for DLT intubation resulted in a longer intubation time, increased intubation difficulty, and increased incidence of intubation-related complications. These contradictory results may be explained by the diversity of operator experience in using GlideScope for DLT intubation. Yi et al. (66) also reported that GlideScope prolonged the DLT intubation time compared with Macintosh, although it improved the exposure of the glottis because the method is more complex.

Wasem et al. (74) found no significant differences between Airtraq and Macintosh regarding intubation time or the number of attempts required for successful DLT insertion; however, a higher incidence of hoarseness was observed with Airtraq. Other studies have also reported that videolaryngoscopes are not superior to the conventional Macintosh laryngoscope for DLT intubation in patients with anticipated normal airways (7, 44, 64, 75, 77).

In a systematic review and meta-analysis, Liu et al. (58) found that videolaryngoscopy provided a higher success rate of DLT intubation at the first attempt and lower incidences of intubation-related injuries and sore throat. However, videolaryngoscopy increased the incidence of DLT mispositioning. According to the performer's experience, the subgroup analysis showed the success rate at the first attempt with a videolaryngoscope was higher than that with a Macintosh laryngoscope for experienced performers. However, this advantage was not observed in novices. Additionally, the time to DLT intubation was comparable between the videolaryngoscope and the Macintosh laryngoscope. However, the reported outcomes were highly heterogeneous, likely due to the different definitions and types of videolaryngoscopes used in the studies.

Kim et al. (78) performed a network meta-analysis of 23 studies. The patients were classified into four groups according to the types of the laryngoscopes: channeled videolaryngoscope, non-channeled videolaryngoscope, videostylet, and Macintosh laryngoscope. They found that most videoscopes showed higher success rates in the first attempt but an increased risk of DLT malposition than Macintosh. Videolaryngoscopes, particularly non-channeled videolaryngoscopes, seemed time-consuming compared to Macintosh. Channeled videolaryngoscope was also associated with a higher risk of oral mucosal injury but did not increase the risk of sore throat.

Compared with the Macintosh laryngoscope, the lifting forces on the base of the tongue were reduced with the videolaryngoscope (15). However, the cardiovascular response following DLT intubation was not analyzed in the meta-analysis. There are three studies on the cardiovascular response following DLT intubation, but the conclusions differ. Hamp et al. (75) found that arterial blood pressure, heart rate, and catecholamine levels were comparable after DLT intubation using Airtraq and Macintosh. Wei and Tian (68) reported that GlideScope induced milder circulatory fluctuations than did Macintosh according to the change in systolic blood pressure. Feng et al. (76) found that Airtraq-guided DLT intubation required a higher EC50 of remifentanil for inhibiting cardiovascular responses compared to Macintosh when induced with a target-controlled infusion of propofol. This indicated that the cardiovascular response during DLT intubation was more intense with Airtraq videolaryngoscopy than with direct laryngoscopy.

Taken together, compared with a direct laryngoscope, the significant advantage of videolaryngoscope-guided DLT intubation is that it improves glottis exposure and increases the success rate at the first attempt. It does not show any advantage in intubation time; moreover, it increases the incidence of DLT mispositioning in patients with normal airways. Thus, videolaryngoscopy is suitable for DLT intubation. However, it is not considered as the first choice for patients with anticipated normal airways, particularly for anaesthesiologists with limited experience in videolaryngoscope-guided DLT intubation.

## Comparison of Different Types of Videolaryngoscopes for DLT Intubation

There are some differences among the different types of videolaryngoscopes. A channeled videolaryngoscope provides an adjacent passage to advance the DLT toward the glottis but limits the ability to manipulate the tube. A non-channeled videolaryngoscope, particularly one with an angulated blade, requires a stylet preshaped to follow the curve of the blade, and the extreme angulation of these blades may complicate tube delivery into the trachea. Eight studies have compared videolaryngoscopes for DLT intubation, mainly focusing on the comparison between channeled and non-channeled, such as Airtraq vs. GlideScope or McGrath Series 5 (Table 2). There is no consensus regarding which videolaryngoscope is optimal for DLT intubation.

**Table 2:** Random controlled studies comparing different types of videolaryngoscopes for double lumen tube intubation.

**References**	**Groups**	**Patients' characteristics**	Main outcomes^*^
Yi et al. (62)	Airtraq (*n* = 36) GlideScope (*n* = 35)	Predicted normal airways	Airtraq provides shorter intubation time, better glottic view, less MAP, HR than GlideScope
El-Tahan et al. (67)	Airtraq (*n* = 21) GlideScope (*n* = 21) Non-channeled KVL (*n* = 21)	Manikin: simulated easy and difficult airways	In easy airway, GlideScope provides shorter intubation time and less intubation difficulty scores than Airtraq and KVL; In difficult airway, KVL had higher intubation difficulty scores than GlideScope and Airtraq
El-Tahan et al. (64)	Airtraq (*n* = 35) GlideScope (*n* = 34) Non-channeled KVL (*n* = 32)	Predicted normal airways	Compared with GlideScope, the Airtraq resulted in shorter times for DLT intubation, a lower score of difficult intubations and fewer optimization maneuvers
Wan et al. (63)	Airtraq (*n* = 45) McGrath Series 5 (*n* = 45)	Predicted normal airways	Airtraq provides shorter intubation time than McGrath Series 5
Belze et al. (79)	Airtraq (*n* = 36) GlideScope (*n* = 36)	Predicted or known difficult airway	No significant difference in outcomes
Ajimi et al. (80)	Airtraq (*n* = 30) AWS-200 (*n* = 30)	Predicted normal airways	Airtraq provides shorter intubation time than AWS-200
Chang et al. (81)	Lighted Stylet (*n* = 32) GlideScope (*n* = 32)	Predicted normal airways	Lighted stylet allowed easier advancement of the DLT toward the glottis and reduced time for DLT intubation compared with GlideScope.
Huang et al. (6)	C-MAC(D) (*n* = 30) GlideScope (*n* = 30)	Predicted normal airways	C-MAC(D) provides better glottic view, shorter intubation time and less difficulty score of DLT delivery and insertion than GlideScope

* *This table just lists the significantly different outcomes between groups. If the outcomes were comparable between groups, they were not listed*.

*DLT, double lumen tube; HR, heart rate; MAP, mean arterial pressure*.

Consistently, Yi et al. (62), El-Tahan et al. (64), and Wan et al. (63) reported that Airtraq provided more rapid intubation of DLT than GlideScope or McGrath; however, the success rates at the first attempt, intubation difficulty score, DLT malpositioning, and intubation-related complications were comparable. The authors attributed the longer intubation time to the use of a molded stylet and a steering technique. These results are different from a manikin study (67) in which they found a longer intubation time and greater intubation difficulty with Airtraq than with GlideScope. The authors attributed the contrary findings to the diversity of prior operator experience and the inherent problems (e.g., high resistance because of the simulator material) in all manikin studies. In addition, El-Tahan et al. study (64) has shown that the Airtraq had an advantage in avoiding tracheal cuff ruptures during insertion of the DLT, compared with GlideScope and non-channel KVL, but a network meta-analysis showed the oral mucosal damage occurred most frequently with the channeled videolaryngoscope (78).

Both Airtraq and AWS-200 are channeled videolaryngoscopes. A recent study (80) described that DLT intubation was quicker with Airtraq than with AWS-200. The authors explained that the difference was attributed to the shape and special treatment of the side channel with Airtraq.

Both GlideScope and C-MAC(D) are non-channeled videolaryngoscopes. The blade of GlideScope has an angulation of 60°, while the C-MAC(D) videolaryngoscope has an angulation of 40°. In the study by Huang et al. (6), the DLT insertion time was shorter with C-MAC(D) than with GlideScope. However, they found no differences in the success rate, DLT malposition, and incidences of intubation-related complications.

All of the above studies were performed in normal airways. Belze et al. (79) enrolled elective thoracic patients with a predicted difficult intubation score of ≥7 and compared the efficacy of GlideScope and Airtraq for DLT intubation. They found no differences in the overall success rate, visualization of the glottis, intubation time, and side effects between the two videolaryngoscopes. Thus, the success rate of DLT intubation for difficult airways is not dependent on the videolaryngoscope used.

Taken together, current evidence does not indicate which videolaryngoscope is superior to others for DLT intubation in glottic view, intubation success, first attempt intubation, and incidence of mispositioning. Although a slight difference in intubation time can be observed between different videolaryngoscopes, there was not much clinical significance.

## Choices of Videolaryngoscope in Managing Difficult Airways in Thoracic Surgery

**Figure 2:**
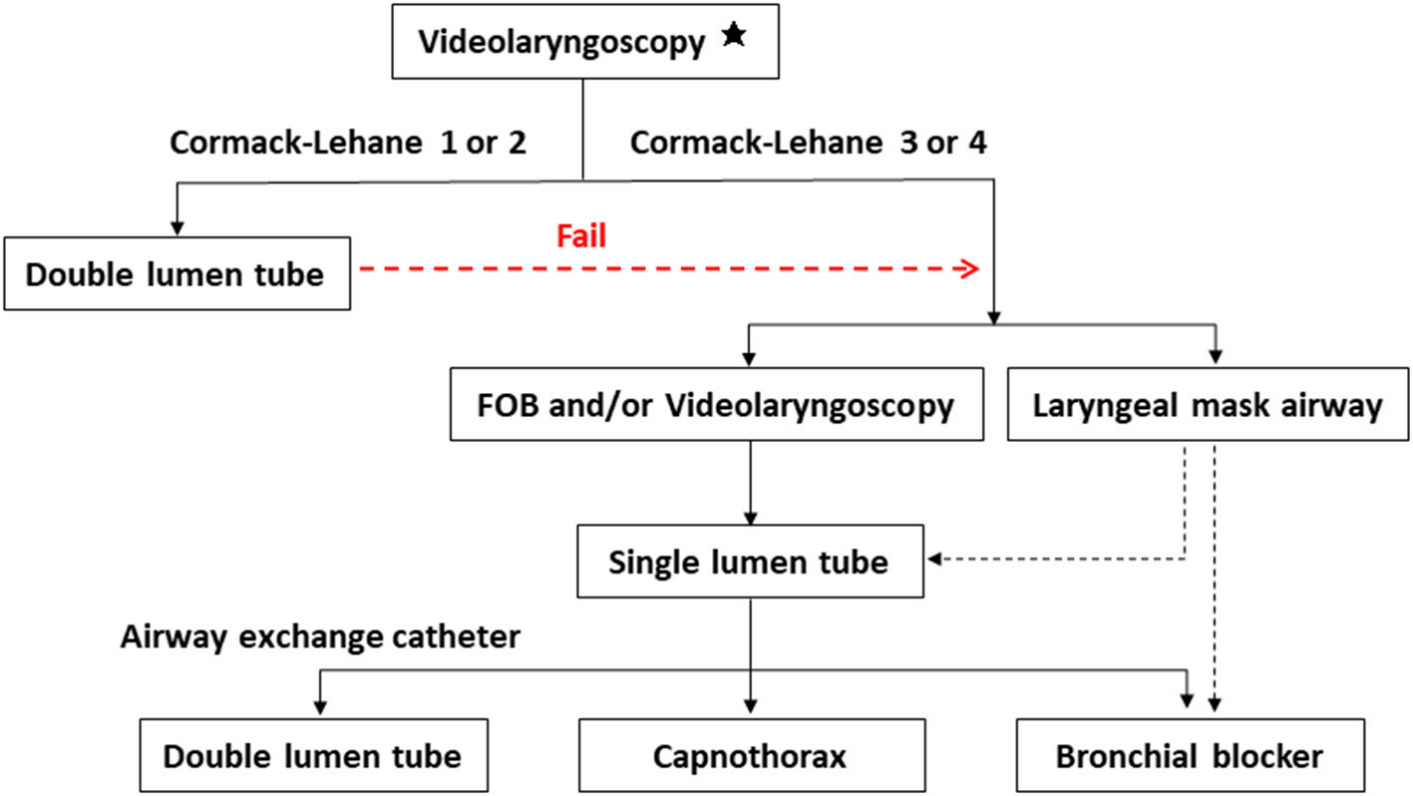
Several strategies to achieve one-lung ventilation in difficult airways using videolaryngoscopes. ^*^Backward, upward and rightward pressure of larynx (BURP) maneuver is used to improve glottic exposure if required. Using videolaryngoscopy, the glottic view is determined according to Cormack-Lehane classification. Grade 1, most of the glottis is visible; grade 2, partial glottis is visible; grade 3, only the epiglottis is visible; grade 4, not even the epiglottis can be seen. If the glottic view is adequate, a double lumen tube is placed with the guide of videolaryngoscope. In case of failure or inadequate glottic view, a single lumen tube is placed with FOB and/or videolaryngoscope, or the patient is secured with a laryngeal mask airway. Then one-lung ventilation is achieved through exchange of a double lumen tube over an airway exchange catheter, inserting a bronchial blocker, or capnothorax. FOB: fibreoptic bronchoscopy.

Difficult airways can be divided into the following five categories: difficult facemask or supraglottic airway (SGA) ventilation, difficult SGA placement, difficult laryngoscopy, difficult tracheal intubation, and failed intubation (26). In thoracic surgery, difficult airways are very challenging. The first challenge lies in securing the airway and maintaining oxygenation; the second involves achieving one-lung isolation using DLT or a bronchial blocker (BB) (3). Due to the distinct advantages of videolaryngoscopy, several options can be considered for managing difficult thoracic airways with videolaryngoscopes (Figure 2).

### Videolaryngoscope-Guided DLT Intubation

Several case reports (40, 46, 49, 60) have reported that videolaryngoscopes successfully managed the DLT intubation for patients with difficult airways (Table 3). Lin et al. (89) reported that in cases of difficult laryngoscopy and failed DLT intubation with Macintosh, using the CEL-100 videolaryngoscope improved the glottic view with a ~90% success rate for DLT insertion. A recent randomized clinical trial (71) reported that the McGrath videolaryngoscope provided a better glottic view and decreased the overall DLT intubation difficulty score in patients with simulated difficult airways through manual in-line stabilization of the cervical spine. Belze et al. (79) reported no difference in the overall success rates, glottic visualization, intubation time, and side effects between GlideScope and Airtraq for DLT intubation in difficult airways.

**Table 3:** Videolaryngoscope-guided double lumen tube intubation in difficult airways.

**References**	**Device**	**Patients' characteristics**	**Awake (yes/no)**	**Note**
Hernandez and Wong (40)	GlideScope	Anticipated difficult airway (BMI 34, Mallampati 3)		
Chen et al. (82)	GlideScope	Unanticipated difficult airway		GlideScope guided SLT placement, then exchange DLT with AEC
Onrubia et al. (83)	GlideScope	Predicted difficult airway and broncho aspiration risk	Yes	
Suzuki et al. (46)	Pentax AWS	Cormack-Lehane grade 2b with Macintosh 4 blade		Remove the back plate of the tube channel
Poon and Liu (84)	Pentax AWS	Two patients with difficult conventional laryngoscopy		AWS guided placement of AEC or bougie first
Sano et al. (85)	Pentax-AWS	A patient with severe rheumatoid arthritis with restricted mouth opening and head tilting		With the newly developed Intlock for DLT
Salazar Herbozo et al. (86)	Airtraq	Two expected difficult patients	Yes	
Ajimi et al. (60)	Airtraq	A case of intubation difficulty (micrognathia)		With the universal adapter for smartphones
El-Tahan et al. (49)	Non-channeled King Vision	A morbidly obese patient (BMI 41.7), a short thyromental distance and a limited mouth opening		
Imajo et al. (87)	Broncho fiberscope combined with McGRATH MAC	Previous upper cervical spine surgery, a small jaw and restricted mouth opening		
Goh and Kong (88)	McGrath	A known difficult airway, bronchopleural fistula, and acute respiratory distress syndrome	Yes	
Lin et al. (89)	CEL-100	Failed DLT intubation with Macintosh		48 Cases
Belze et al. (79)	GlideScope vs.Airtraq	Patients with a predicted difficult intubation score of at least 7		RCT
Yoo et al. (71)	McGrath vs. Macintosh	Patients with a simulated difficult airway		RCT

*AEC, airway exchange catheter; BMI, body mass index; DLT, double lumen tube; SLT, single lumen tube; RCT, randomized controlled trial*.

Based on a detailed evaluation of the airway history, physical examination, and additional evaluation in cases of anticipated difficult laryngoscopy or difficult intubation, if a DLT is applicable, the performer may reasonably choose a videolaryngoscope as a primary intubation tool. The choice of videolaryngoscope is based on individual experience, preference, and availability. Studies have demonstrated a correlation between intubation success and the operator's proficiency in the device rather than the device used (58, 79).

If the glottic view is adequate, but guiding the tip of the DLT into the glottis or advancing the tube pass through the glottis is difficult, videolaryngoscopy combined with FOB may be an effective approach. In the study by Imajo et al. (87), Lai and Wu (90), a fibreoptic bronchoscope was used as a stylet and placed through the bronchial lumen. Under videolaryngoscopy observation, they guided the tip of the tube to the glottis using FOB and facilitated DLT intubation. This hybrid method has an additional benefit of real-time visualization of all procedures, allowing the anaesthesiologist to ensure successful and safe intubation. For patients with restricted neck movement or limited mouth opening, a videostylet may be a useful alternative tool for DLT placement (91, 92).

Although awake fibreoptic DLT intubation is a good airway management option for an anticipated difficult airway, it has several disadvantages, such as the difficulty of the technique, and anatomical structures, such as upper airway soft tissue resistance, which can make it difficult to intubate. Previous reports (83, 86, 93) have described several alternatives to FOB, such as GlideScope, Airtraq, and videostylets that can be effectively used in patients with difficult airways for awake DLT intubation.

### Videolaryngoscope-Guided SLT Intubation

It is more difficult to intubate a DLT than a standard SLT. For anaesthesiologists with limited experience in thoracic anesthesia, although videolaryngoscopes can improve glottic exposure in patients with difficult airways, videolaryngoscope-guided DLT intubation can fail (82). Videolaryngoscope-guided SLT placement is familiar to most anaesthesiologists. In this situation, the first choice is to secure the airway with an SLT under videolaryngoscope guidance. Otherwise, the combined use of videolaryngoscopy and FOB can be used for SLT intubation (94). Therefore, we must now consider how to achieve lung isolation. In the following section, several strategies are considered for lung isolation after successful SLT insertion.

#### SLT Exchange for DLT Using an Airway Exchange Catheter

Airway exchange catheters (AECs) are an important airway-assisted tool for thoracic anesthesia. For patients with difficult airways who require one-lung ventilation, one commonly used option is to intubate with an SLT and then exchange it with a DLT over an AEC. If postoperative ventilation is necessary, the AEC is also used to exchange the DLT for an SLT.

Chen et al. (82) reported a case of an unanticipated difficult airway in which GlideScope-guided DLT intubation failed in two attempts, while the SLT was successfully inserted on the first attempt. The DLT was then successfully placed over an AEC under the guidance of GlideScope. Poon and Liu (84) described using an AEC alongside AWS-S100 guidance for DLT placement in two cases of difficult laryngoscopy. Although the tracheal tube guiding channel of the disposable rigid blade (PBlade) used in this study cannot accommodate DLTs, Airway Scope can guide bougies or AECs into the trachea and then railroad a DLT over them.

McLean et al. (95) reported a failure rate for exchanging an SLT with DLT of 40%. Tube impingement on the arytenoids or epiglottis is often encountered during the exchange of the DLT over an AEC. The fit of the AEC in the DLT is vital and should be checked before use (82). Further, videolaryngoscopy can be used to visualize the railroading process of the DLT over an AEC. It can also lift the supraglottic tissue to avoid resistance while advancing the DLT into the trachea. Mort and Braffett (96) compared conventional and videolaryngoscopes for ETT exchange in high-risk, difficult airways; they observed that videolaryngoscopy-based ETT exchange over an AEC provided efficient and timely ETT passage and fewer attempts due to improved glottic visualization.

Although McLean et al. (95) reported that the failure rate for postoperative DLT to SLT exchange was 0%, this result should be interpreted cautiously. If DLT placement is extremely difficult, it can be continued to maintain ventilation after surgery until the patient is fully awake. Suzuki et al. (97) reported that using two AECs reduced the risk of tube impingement into the trachea during DLT to SLT exchange.

#### SLT With BBs

Tube exchange has a failure risk not only from DLT to SLT, but also from SLT to DLT. This could also be associated with pneumothorax (95). It is more convenient to use BBs in such cases, another commonly used option for one-lung ventilation (98). BBs are advantageous when a DLT cannot be used, such as in pediatric patients undergoing thoracic surgery, in whom only nasotracheal intubation is possible, and patients with tracheal tumors or abnormalities. Another advantage is that BBs can provide a selective block of the pulmonary segment and postoperative tube exchange is unnecessary.

There are two methods for BB placement: intraluminal and extraluminal. Briefly, in intraluminal placement, the BB is typically inserted into the lumen of the SLT, along with an FOB to guide the BB to an optimal position. Thus, intraluminal placement of BBs requires a large SLT diameter. Intraluminal BB placement seems easy after SLT insertion, but it is difficult to control the BB and FOB simultaneously, even when a large tube is used (99).

Extraluminal BB placement has been described in adults and children (100, 101). Templeton et al. (99) reported that extraluminal BB placement was safe, adequate to excellent surgical exposure, and faster than intraluminal placement. However, it may be difficult to place the BB in cases of poor glottic exposure using the Macintosh laryngoscope. Recently, several reports described successful extraluminal BB and SLT placement via videolaryngoscopy (102, 103). As the BB is very thin and long, it is difficult to control the direction during extraluminal placement under videolaryngoscopy. A guiding tube can be self-made from a routine tracheal tube using an Airtraq videolaryngoscope (103). In children aged <2 years, a 5F Arndt BB was bent at a 35–45° angle at 1.5 cm proximal to the balloon when using Storz C-MAC (104). These tips are very useful for extraluminal BB placement under videolaryngoscopy.

The VivaSight™ SLT, in combination with a BB, is a new method for one-lung ventilation. VivaSight™ SLT is a new generation ETT that incorporates a high-resolution imaging camera and a light source at its tip. It has been reported for BB placement without the aid of FOB and can provide real-time and continuous monitoring of the BB position (105–107).

Rapid lung collapse with a BB is not associated with the type of device used but with the method of use (108). For example, when using an F_I_O2 of 1.0 before one-lung ventilation, an apnoeic period of 30–60 s at the time of the pleural incision by disconnection of the breathing circuit and transient deflation of the BB balloon results in rapid lung collapse (109).

#### SLT With Capnothorax

Traditionally, lung isolation offers excellent surgical exposure during thoracic surgery. However, some complications are associated with one-lung ventilation, such as hypoxemia, bronchoalveolar injury, and postoperative pulmonary complications (110, 111). SLT intubation and CO_2_ insufflation of artificial pneumothorax were introduced into thoracoscopy very early. In 1994, Wolfer et al. (112) studied the effects of CO_2_ insufflation on haemodynamic parameters during thoracoscopy and reported promising results. SLT intubation and CO_2_ insufflation have been previously described in various thoracoscopic procedures (113, 114), including thoracoscopic esophagectomy (115, 116), and have proven to be feasible, efficient, and safe. SLT intubation and CO_2_ insufflation can be an alternative to one-lung ventilation for minimally invasive thoracic surgery, particularly when expertise for DLT placement is unavailable or when an operation is very short and simple, such as pleural effusion drainage and pleural biopsies (117). Yeh and Hsu reported an alternative method to achieve lung isolation using artificial pneumothorax under spontaneous breathing with ETT placement in patients with limited mouth opening (118).

When dealing with unanticipated difficult airways, if videolaryngoscopy fails, a laryngeal mask airway or surgical airway can be considered to secure the airway (5). An SLT can be inserted via the intubating laryngeal mask airway, and then a BB can be used to achieve one-lung ventilation. Alternatively, a BB can be directly inserted via the laryngeal mask airway (119, 120). For patients with tracheostomy, shortened DLTs or BBs can be considered to achieve lung isolation (2, 121).

## Conclusion

Videolaryngoscopy plays an important role in thoracic airway management. Either non-channeled or channeled videolaryngoscope is suitable for DLT intubation. It can improve glottis exposure and increase the success rate at the first attempt. However, it has no advantage in saving intubation time and increases the incidence of DLT mispositioning for patients with normal airways. Thus, it is not considered as the first choice for patients with anticipated normal airways. Current evidence does not indicate which videolaryngoscope is super to another one for DLT intubation. The choice of videolaryngoscope is based on individual experience, preference, and availability. For patients with difficult airways, videolaryngoscope provides multiple options to achieve one-lung ventilation. Due to the distinct advantages in glottic view, videolaryngoscope-guided DLT intubation is a primary and effective method. Nevertheless, it requires training, particularly for novices and anaesthesiologists with limited experience in videolaryngoscope-guided DLT intubation. In case of failure, videolaryngoscope-guided SLT intubation can be achieved because it is familiar to every anesthetist or combined with the aid of fibreoptic bronchoscopy. Placement of a DLT over an AEC, inserting a BB via an SLT, or capnothorax can be selected for lung isolation.

## Author Contributions

WY and CZ: conception or design of the work. ML and WY: literature review and draft of the manuscript. CZ and AL: critical revision of the manuscript. WY: take responsibility for data integrity and accuracy of the data analysis. All authors contributed to the article and approved the submitted version.

## Funding

This study was supported by a grant from the National Natural Science Foundation of P.R. China (No. 82171228 to WY) and a grant from China National Key R&D Program (No. 2020YFC2009002 to AL).

## Conflict of Interest

The authors declare that the research was conducted in the absence of any commercial or financial relationships that could be construed as a potential conflict of interest.

## Publisher's Note

All claims expressed in this article are solely those of the authors and do not necessarily represent those of their affiliated organizations, or those of the publisher, the editors and the reviewers. Any product that may be evaluated in this article, or claim that may be made by its manufacturer, is not guaranteed or endorsed by the publisher.
